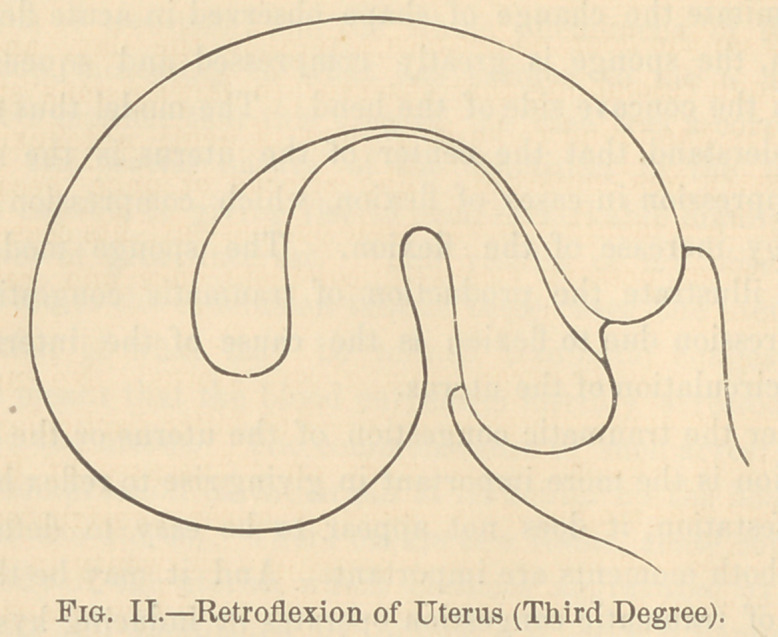# The Exciting Cause of Attacks of Hysteria and Hystero-Epilepsy

**Published:** 1883-02

**Authors:** Graily Hewitt

**Affiliations:** London


### Selections.

### THE EXCITING CAUSE OF ATTACKS OF HYSTERIA AND HYSTERO-EPILEPSY. By GRAILY HEWITT, M. D., London.

Hysteria is a subject which has often and repeatedly attracted the attention of physicians. Hitherto it cannot be said to have been thoroughly elucidated. In attempting to deal with so wide and important a subject, and in offering a contribution towards the settlement of certain vital questions relating to hysteria, I trust I am not guilty of too great a presumption.

In the first place, I desire to present a synopsis of the subject of hysteria, in order that I may be able more clearly to define the position which my observations have induced me to take on the etiology of hysteria.

Synoptical view of the etiology of hysteria and hystero-epilepsy:—

A. Condition of nervous centers. 1. Unduly impressional. *a.* Emotionally. *b.* By reflex action. 2. Actually diseased.

B. Presence of peripheric irritation. 1. In uterus. 2. In ovary. 3. Elsewhere.

Hysteria may be, according to this synoptical view, either "centric" or "peripheral" in origin. It is also evident that there is no absolute incompatibility in the two views.

It has long been a matter of belief—dating, in fact, from the Hippocratic era—that irritation in the generative organs plays a very important part in the production of hysteria.

Gynæcologists, who have most knowledge of the truth of this idea, have not, however, up to the present time been able to define precisely the *modus operandi* and the exact nature of the irritation in question.

Assuming that the hysterical manifestions, convulsions, etc., seen in women are brought about by the irritations of the sexual organs, the initial difficulty presents itself as to which of the two organs, the ovary or the uterus, is mainly responsible.

So far as hysterical convulsions and hysterical symptoms are concerned, the opinion held by some authors—Negrier and Charcot—is in favor of the view that the ovaries are the *point de départ.*

The observations of Charcot are most interesting, and the phenomena of the hysterical paroxysm have been described by him, and still more recently by Richer,* in a manner which leaves little to be desired so far as the outward manifestations, convulsions, spasms, anæsthesia, paralysis, temporary intellectual disturbances, etc., are concerned. And these delineations are also most complete in regard to the manner in which the manifestations in question are capable of being modified or influenced by the action of *external* agencies. The ebullition, as it may be termed, has, in short, been pictured in a most graphic manner.

Circumstances have led me to investigate the various hysterical manifestations observable, from an etiological point of view. I had no predispositon to take any particular view of the matter, and it was only by repeated observation that I became convinced that the uterus is generally in a state of irritation in cases where these manifestations are observed; thus, in fact, confirming the more ancient theory of the subject. And I was induced to take this view of the influence (etiologically) of the uterus from the circumstance that in cases where the two conditions were conjoined—viz., uterine irritation and liability to attack—the attacks always appeared to cease on removing the irritation. In fact, experience revealed to me that in the course of treating the disorder of the uterus, the liability to hysterical attacks ceased. Further observation showed that the peculiar irritation productive of hysterical symptoms and attacks was always one and the same —viz., a flexed and distorted state of the uterus. Since I first became aware of this relation I have omitted no opportunity

* "Etudes Cliniques sur l'Hystéro-épilepsie ou Grand Hystérie." Par Dr. Paul Richer, Paris: Delahaye. 1881.

which has occurred to me for verifying and repeating the observation. Cases of this kind now referred to, do not present themselves with great frequency; cases of marked hysterical paroxysms, so far as my experience goes, are not very common, but during the last ten years, during which I have been testing the matter in question, several instances have fallen under my notice; and as yet the facts I have collected are strictly confirmatory of the truth of the above generalization.

There appear to be two classes of cases :—

1. Those in which the attacks are induced primarily by some strong emotion—the reception of distressing news, a fright of any kind, a severe mental shock, etc. Here the operation of the causes is a direct action on the central nervous system, which in such cases may or may not be weakened in some way, and predisposed, or not, to be affected by an excitement acting from without. These cases are undoubtedly met with in practice, but they seem to be rather rare.

2. Those in which the attacks are induced primarily by a reflex disturbance from within, and quite distinctly so. This class of cases is numerically far more frequent than those classed in the foregoing list. They include cases in which the hysterical manifestations are severe, and more or less constantly liable to occur.

Now, the evidence which I have been able to collect, to me convincingly shows that the reflex irritation causing these attacks and other hysterical manifestations is an irritation having its seat in the uterus, and that the particular irritation most potent in producing the reflex disturbance is flexion of the uterus. This view is one which I expressed about twelve years ago.

In the course of my professional experience, I have only met with cases which seemed to be cases of hysteria produced in the reflex manner, and I have seen none in which hysteria of a severe character has been brought about emotionally. I do not deny the existence of the latter class of cases (certain of M. Charcot's cases, for instance), but it so happens that I have seen none. On the other hand, I have met with many cases coming under the former category, and in such cases, the uterus was found to be the cause of the symptoms; the facts of the cases, the results of treatment and the whole phenomena of the cases in question, indicating in what has seemed to me a most unmistakable manner that this view of the case was a correct one.

What the precise nature of the condition of the uterus is which is capable of giving rise to such remarkable manifestations is a matter of great interest. The results of my observations have led me to the conclusion that in these cases the uterus is in a condition of what may be termed traumatic congestion, by which is meant that the blood current is forcibly arrested in the tissues of the uterus. The common cause of such arrest in these cases is compression of the organ at its center by the bending or flexion of the uterus. There occurs as a result, acute congestion of the body of the uterus, which becomes aggravated by certain movements and diminished by others. Whence it happens that exertions capable of increasing the flexion, are found to bring on the attacks or other hysterical manifestations, while, as a rule, rest and the horizontal position are equally potent in removing them or in preventing their occurrence.

The word "traumatic" seems suitable as explaining the nature of the congestion present under these circumstances.

The intensity of the traumatic congestion present in different cases appears to vary, but its main characteristics seem to be the same in all instances that I have observed. And the worst and the severest cases of hysterical convulsions have been those in which the degree of traumatic congestion of the uterus was actually greatest.

There is another etiological moment present—viz., the compression of the nervous filaments of the uterine tissue at the precise spot where the flexion compression is greatest. When the uterus is forcibly flexed, such compression occurs.

I have here the model of a section of the uterus constructed in sponge.* The model is six times the normal length of the uterus, but the thickness of the walls and the due relation of the parts are carefully preserved. The model is constructed in order to exhibit the effects of acute flexion of the uterus on the uterine tissues. It will be observed that when the sponge uterus is bent

* The model was here exhibited by the author, and the action of flexion on the uterus illustrated by its means.

so as to imitate the change of shape observed in acute flexion of the organ, the sponge is greatly compressed and squeezed together on the concave side of the bend. The model thus enables us to understand that the center of the uterus is the seat of great compression in cases of flexion, which compression is increased by increase of the flexion. The sponge model also serves to illustrate the production of traumatic congestion, for the compression due to flexion is the cause of the interference with the circulation of the uterus.

Whether the traumatic congestion of the uterus or the flexion compression is the more important in giving rise to reflex hysterical manifestation, it does not appear to be easy to determine. Possibly both moments are important. And it may be that the presence of traumatic congestion operates in inducing hysterical phenomena by virtue of the compression of the uterine nerves in those parts of the uterus which are actually the seat of the congestion.

The accompanying drawings represent flexions of the uterus severe in degree. Fig. 1, shows a third degree of anteflexion of the uterus. Fig. 2, represents the uterus in a case of retroflexion in the third degree. The seat of the compression is principally the wall of the uterus on the concave side of the flexion.

I adduce in support of the views now enunciated a series of eighteen cases, arranged in chronological order, observed by me

FIG. I.—Anteflexion of Uterus (Third Degree).

FIG. II.—Retroflexion of Uterus (Third Degree).

during the ten years from 1870 to 1880. I have observed other cases also, of which records have not been kept. The following series are all of which I have kept records. There are six cases in which the uterus was retroflexed, and twelve in which anteflexion was observed.

CASE I. Chronic retroflexion. Severe hysterical attacks.—Mrs. —— had been liable to frequent severe hysterical attacks, after which she usually remained in a state of quasi-insensibility for some time, ever since her first confinement, which occurred upwards of twelve years previously. Latterly severe sickness had occurred. The uterus was found acutely retroflexed. There was an absolute cessation of the hysterical attacks from the time the treatment of the retroflexion was commenced.

CASE II. Acute anteflexion of the uterus. Almost entire suspension of menstruation for two years. Severe hysterical attacks.—The following is an abstract of the case which is more fully published in the third edition of my work on "Diseases of Women." The patient was single, æt, nineteen, a dressmaker. Two years ago became attacked with "hysterics," at first severe, afterwards less so. On one occasion she lost her voice for five months. Has had lately a peculiar cough. Menstruation only once in the two years. While in the hospital had several severe hysterical attacks, strong convulsive action and attempts to beat her head on the floor, sometimes several in the day, and a peculiar cough resembling that observed in laryngismus stridulus. The uterus was found to be in a state of acute anteflexion. A stem pessary was employed. The attacks at once became less frequent. In a month she was made out-patient; fits ceased. Two months latter pessary removed, complete cure and return of menstruation.

CASE III. Acute anteversion of the uterus. No menstruation. Severe epileptiform attacks.—For fuller particulars of this case see third edition of my work on "Diseases of Women." The patient was single, æt. seventeen; never menstruated. For ten weeks has had fits, as many sometimes as twenty in the day. In service since age of ten years. Pains in hypogastrium and frequent micturition for four months. Uterus anteverted. Sound easily introduced. Cradle pessary introduced. A month later the fits had become reduced in frequency, and she left the hospital. Menstruation appeared about two months after commencement of treatment and was followed by a complete cessation of the attacks.

CASE IV. Acute anteflexion of the uterus probably of one year's duration. Convulsive attacks occurring frequently during that time.—The patient was married, thirty years of age, had one child four and a half years ago. Health tolerably good till one year ago. Six weeks nursing a sick child appears to have made her ill. The illness began with an attack consisting of slight loss of consciousness for a moment, then convulsions. Since that time has had two attacks—two or three a day as a rule; the attacks last a short time, are not accompanied with loss of consciousness as a rule, and during the last three months have become more intense; menstruation had also ceased for three months, but has just occurred again once. The uterus was found acutely anteflexed. A cradle pessary was applied, the sound used to straighten the uterus. The attacks became at once reduced in frequency and intensity. During the first four days had altogether eleven attacks; during the succeeding ten days only five attacks; altogether she was under observation for seven weeks; the attacks latterly only occurred once in two or three days, and were very slight, while menstruation had occurred a second time rather profusely.*

CASE V. Retroflexion of the uterus. Hysterical attacks following exertion.—Mrs. ——, æt. nineteen, married fourteen months. Has had no child. Suffers from hysterical attacks, and her medical attendant believed her to be affected with retroflexion of the uterus.

It appears that four years before marriage she had a severe attack of scarlet fever, which left her so weak that she did not walk for one year, and then began with crutches. Since recovering from this attack she has been liable to what are termed hysterical attacks, following any exertion.

Menstruation is profuse and too frequent. The uterus is soft to the touch, very distinctly retroflexed. A pessary was applied.

The patient completely recovered, and had a child two years afterwards.

CASE VI. Acute anteflexion. Severe hysterical attacks.—Mrs. ——, æt. thirty-four. Has been married fifteen years; has had no children. Menstruation always painful. Has had bearing-down for years. Ten years ago had St. Vitus's dance, not severely; but has occasional symptoms on and off, such as nervousness for an hour or two when excited. Six months ago had been nursing, for five months severely, and began to feel excessive bearing-down and strangury, became insensible for a week, and urine had to be drawn artificially. Had also acute pain in abdomen and hypogastric region, the difficulty in passing water continuing. She had severe convulsions at intervals during the time. Ever since this time she has had severe attacks of what are termed "strong hysterics" after any slight fatigue. Uterus in a state of acute anteflexion. A cradle pessary was applied. Relief. Later history not known.

CASE VII. Retroflexion of the uterus. Hysterical attacks.—E. J., a cook, single, æt. twenty-six. Three years ago was under treatment for uterine affection. Has suffered for some time now from hysterical attacks, which last for about twenty minutes, and

* Fuller particulars of this case in Lancet, August 7, 1875.

during which she becomes unconscious. The last attack came on during the singing in church, and she had to be carried out.

Uterus retroflexed. A pessary applied. Cure.

CASE VIII. Slight anteflexion of the uterus. Attacks of convulsions. Mrs. ——, æt. thirty-three, had four children, the last born six years of age. Six months ago had a convulsive seizure, following a course of nursing and over-exertion. The convulsions produced a kind of opisthotonos. She was conscious throughout, but could not move for ten days. Since this attack has occasional twitchings. No sickness. Easily tires from short walks. Uterus a little anteflexed. Sound enters with difficulty. Treatment, rest. Result, favorable.

CASE IX. Acute anteflexion of the uterus. Suppression of menstruation. Severe hysterical attacks.—Miss ——, æt. twenty. Has always been weak and delicate. Menstruation began at twelve.

Two years ago she bathed in the sea just before the time for the period, and it did not, consequently, occur. She became very ill, and menstruation did not occur for three months. Since that time she has been liable to severe hysterical attacks, and to frequent threatenings of attacks. There was a further catching of cold five months ago, and the menstruation has not occurred since, with one exception.

The uterus was found very low down in the pelvis, and anteflexed. A cradle pessary was employed. The hysterical attacks ceased, but the patient remained for some time in a weak condition. Finally, restoration to health. The hysterical symptoms did not recur.

CASE X. Retroflexion of the uterus. Hysteria.—Miss ——, æt. forty-one. Had a fall from a horse twenty years ago, and has been ill ever since. Treated for hysteria for a long time. It was discovered, nine months ago, to be a case of retroflexion, by Mr. Palmer, of Nayland, Colchester, who has nearly succeeded in restoring the uterus to its proper place, and she is now much better.

CASE XI. Acute anteflexion of the uterus. Severe hystero-epileptiform attacks.—Mrs. ——, æt. twenty-one. Married three years. Ill since six months after marriage. Is subject to severe hystero-epileptic attacks. These chiefly occur after sitting upright, as at meals. They are very severe, and the general disturbance is very acute.

The uterus is in a state of acute anteflexion and much tilted forwards. There is very great tenderness of the epigastrium and of the back, particularly at three special spots.

The flexion and displacement were treated by the sound and a cradle pessary. The attacks were relieved at once, and have not returned since.

CASE XII. Acute anteflexion of the uterus. Severe convulsive attacks.—Miss ——, æt. thirty-eight. Out of health one year. Had an attack of bronchitis, on recovering from which she had a succession of severe nervous attacks, on one occasion being for five or six hours unable to speak, move, or show any signs of life, but was all the time conscious. There were many other severe attacks. For three or four months could not sit up one hour, though she could walk a little. Has not improved the last three months. To quote the patient's own description:—"There is constant pain in the back, almost constant sickness or nausea, occasional violent retching brought on by walking or even talking. Any exertion of mind or body produces clinching of the hands, and a horrid feeling all over the back and back of the head. Menstruation regular, but extremely painful, and inability to move at these times increased. Feels very often faint, and a sensation then begins in the brain. She feels that she cannot speak, and is very unlike herself. On recovering, feels as if she had been some one else all the time, or as if she had two selves, one quiet and sane, the other idiotic."

Severe anteflexion of the uterus. Treated by a cradle pessary. Complete cure.

CASE XIII. Acute anteflexion of the uterus. Severe convulsive attacks just previous to menstrual periods.—Miss ——, æt. twenty-eight. Has suffered from severe convulsive attacks since menstruation commenced. These attacks appear generally just previous to menstruation. They have been considered due to disease of the brain.

The attacks are of the following kind:—The eyes become fixed on the ceiling, the teeth clenched, the back arched and rigid, the limbs also contracted and set. There is incapability of speaking, but the patient knows what is going on. The skin is deadly cold. The attacks last from an hour to an hour and a half. The patient was found to be suffering from acute anteflexion of the uterus. She was treated for this by a cradle pessary and occasional use of the sound. After three months the attacks had become greatly lessened in frequency. Half a year elapsed before the patient was next seen. The attacks had disappeared. A slight sensation of faintness only was occasionally observed at times. A year later, still free from attacks. The anteflexion of the uterus was difficult to cure in this case, but the final result satisfactory.

CASE XIV. Retroversion and slight flexion of the uterus. Convulsive attacks about menstrual periods.—Miss ——, æt. twenty-nine. Four years ago began to suffer from convulsive attacks which always came on about the second day of the menstrual period. She remains insensible about half an hour (once in two days) after the attack. Has had five attacks. Has had much exertion in lifting and nursing. Uterus markedly retroverted and a little flexed. Treated by pessary. Cure.

CASE XV. Acute retroflexion of the uterus. Severe hysterical attacks. Mrs. ——, æt. thirty-eight. Has had no children. About one year ago began to have severe hysterical attacks, with screaming and much excitement. Occasionally every word excites the sensation of an attack coming on. Formerly could walk well. Walking power now very much more limited.

Uterus acutely retroflexed, extremely sensitive to the touch. Treated by the sound and by a Hodge pessary. One year afterwards she stated that she had had no more attacks, and was in all respects feeling quite well and strong.

CASE XVI. Uterus anteverted.—Hysterical attacks. Mrs. —— æt. thirty. Four children. Hysterical attacks and pain after exertion. Uterus anteverted, wearing a Hodge pessary, the overaction of which has produced anteversion of the uterus, or exaggerated it.

CASE XVII. Anteflexion of the uterus. Hysterical attacks. — Mrs. ——, æt. twenty-four. Three children. Two years ago began to have hysterical attacks, with pains in the head, and dullness. Since last confinement, five months ago, the attacks are more frequent. The patient has a frequent choking sensation. She is obliged to stand a good deal.

Uterus low down, anteflexed; fundus close to symphysis pubis.

Treated by a cradle pessary. Cure.

CASE XVIII. Anteflexion of the uterus. Hysterical attacks and severe sickness. Miss ——, æt. thirty-three. Five years ago lifted a heavy weight, and fell ill in consequence. Two years ago began to suffer from sickness. The sickness has been almost incessant ever since. Dysmenorrhœa also of late. For the last four months has been subject to fits of insensibility. The head feels strange; she lies down and knows no more for some time—once for as long as twenty-four hours. When she returns to herself has much aching of the jaws. Uterus very low down, larger and anteflexed. There is great tenderness over right ovarian region. Very severe and troublesome sickness almost constantly present. Treated by cradle pessary. Great improvement, sickness subsided, attacks ceased. Pessary removed one year and nine months later, when the patient seemed well. Five months later, return of symptoms and re-employment of cradle pessary.

The cases above related, coupled with others which I have seen, but of which I possess no sufficiently good records, have induced me very decidedly to come to the conclusion that it is the uterus which is the seat of the irritation, which issues in the hysterical attack. The manner in which the attacks originated, the circumstances attending the subsequent occurrence of them, the relief, and in many cases the instantaneous manner in which the attacks ceased when the uterus was straightened and put into its proper position in the pelvis—the facts and observations, repeated over and over again have forced this conclusion upon me.

The occurrence of hysterical paroxysms was, in the large majority of cases which I have witnessed and investigated, apparently brought on by some physical exertion. This is the most important circumstance. The importance of it arises from the following considerations:—When the uterus is in a state of flexion, either forward or backward, the act of lifting, or stooping, or over-walking, or standing, has the effect of intensifying the flexion; the uterus is pushed lower in the pelvis, and its curvature becomes exaggerated. This is a fact abundantly borne out by clinical observation. The result of increase of the flexion of the uterus is to increase the congestion; there is in such cases congestion to begin with, but the physical exertion leads to its very considerable aggravation, and when the aggravation reaches a certain point the hysterical attack appears.

On the other hand, by taking measures such as are adapted to prevent the aggravation of an existing flexion—that is to say, by keeping the patient in a horizontal position—the attacks are not found to occur, or, at all events, become much diminished.

Observation shows that the dorsal position prevents hysterical attacks due to anteflexion, but that the prone position is most effective when the case is one of retroflexion. These facts are most interesting. Out of the eighteen cases related, twelve were cases of anteflexion, from which it appears that the most common cause of hysterical attacks is presence of anteflexion of the uterus. One of the principal reasons why the mechanism of the production of the hysterical paroxysm has so long escaped recognition is, I believe, the fact that anteflexion of the uterus has, up to quite a recent period, been hardly allowed a place in nosology. I cannot stay here to explain this latter circumstance; but I take the opportunity of saying that, having for many years closely observed and investigated the mechanical diseases of the uterus, I have long been impressed with the grave nature and frequency of the symptoms to which this variety of distortion and displacement of the uterus is capable of giving rise.

I may be permitted, in conclusion, to make a few remarks on the ovarian theory as to the origin of the attacks, which has of late been so warmly advocated by Professor Charcot.

It is well known to gynæcologists that the ovary is sometimes found to be prolapsed, and can be readily felt in the Douglas' pouch. It is there subjected to great pressure and irritation, and much pain and suffering is found to be present in such cases. These cases would therefore be supposed to be of all other cases in which hysterical attacks should occur, supposing that the ovaries are the principal point of origin. I do not deny that such dislocation of the ovary may cause hysterical attacks ; but I have, at all events, not seen attacks of hysteria in such cases of dislocated ovary, unless accompanied also by acute anteflexion of the uterus. Retroflexion and dislocation of the ovary are not seldom associated.

Further, in the cases of hysteria above related, where flexion of the uterus was undoubtedly present, the ovaries were not found to be particularly sensitive, nor was there evidence of ovarian disease.

The fact that pain is frequently felt in the ovarian region in cases of hysteria, on which much stress has been laid by those who adopt the ovarian theory, is explained by the flexion of the uterus. Having made many observations on this subject, I am able to state that pain in the ovarian region is a very common symptom in cases of uterine flexion. It appears to be due to the fact that the flexion is generally a little to one side, the uterus not being usually bent directly backwards or forwards, but most usually a little to one side or the other.

Two series of facts described by Professor Charcot are adduced by him to support the theory that the ovary is the *point de départ* of the paroxysm in hysteria and hystero-epilepsy.

In the first place, Charcot states that pressure over the lateral hypogastric region has the following effect:—"Pressure there produces not only pain, but a sensation accompanied by all or some of the phenomena of the aura hysterica. Thus, methodical compression of the ovary determines the production of the aura, or sometimes even a perfect hysterical seizure."

In the next place, Charcot states that a more energetic compression is capable of stopping the development of the attack, when beginning, or even of cutting it short when the evolution of the convulsive accidents is more or less advanced.

The method adopted by Professor Charcot to effect the more severe compression is as follows:—

"The patient should be horizontal in dorsal decubitus on the floor, or a mattress. The physician then kneeling on one knee, presses the closed hand, or fist, into that iliac fossa which he had previously learned to regard as the habitual seat of the ovarian pain. At first much force is required to overcome the abdominal muscles. Pressure then produces numerous and noisy attempts to swallow. Consciousness returns almost at the same time. Now the woman moans and weaps, says she feels relief, or that you are hurting her. By continuing the pressure two, three or four minutes, you are almost certain to find all the phenomena of the seizure to disappear as if by magic. When the abdominal resistance is overcome, pressure by the two first fingers is sufficient."*

It may be desirable to consider how far the results of Professor Charcot obtained by pressure, as above described, over the ovarian region, are antagonistic, or the reverse, to the uterine theory above formulated, as to the cause of the paroxysm in hysteria and hystero-epilepsy.

The pressure employed by Professor Charcot is a very forcible pressure made in the hypogastric lateral region, calculated, first of all, to abolish the resistance of the abominal muscles—a resistance considerable in many cases; and secondly, to produce a real compressing influence on the organs which lie in the pelvis. The incidence of this pressure, which is effected by the fist, or by an apparatus specially contrived for the purpose, is rather widely spread, and it is such that it must almost of necessity affect not only the ovary, but first the uterus, and secondly the ovary. Doubtless when the resistance of the abdominal muscles is overcome, the pressure can be more particularly pointed on, or directed towards the ovary, or concentrated on this latter organ. But at the same time it is almost inevitable that the uterus should be greatly affected by this pressure, and must receive a considerable portion of it. Considering for a moment the operation of such pressure on the uterus, the effect might be different, according to the position of the uterus at the time. Thus, if the uterus were much anteverted, the result would, or might be, to push it still lower in the pelvis, and to increase the anteversion; but the action of the pressure would be further to express the blood from the uterine vessels, and to diminish any congestion of

* See New Syd. Soc. trans. of Charcot's "Lectures," p. 27.

the organ existing at the time. If the pressure were made directly behind the pubic bone, the effect might, on the other hand, be such as to push the uterus backwards, and, in the next place, to drive the blood out of its tissues. A further effect of the pressure would be, in any case, to diminish the flow of blood to both uterus and ovaries alike, by the general action of the compressing power on the blood of the pelvis.

So far as I am able to judge, therefore, it would appear that the operation termed ovarian compression is really entitled to be denominated "uterine," quite as much, perhaps even more, than it is to be described as ovarian compression.

But this is not all. Professor Charcot states that slight pressure of the kind above described often brings on pain and symptoms of the hysterical aura—that is to say, the attack is capable of being brought on slight pressure, and relieved by severe pres. sure. All this is quite in unison with the argument which I just advanced, for supposing a version or flexion to exist, the slight pressure above the pubes, such as Charcot describes, would undoubtedly at first intensify the displacement. The slight pressure would temporarily thus so act on the uterus as to induce the attack.

In conclusion, I would express my conviction that the escape from the indefiniteness of view, which up to the present time has characterized the various opinions entertained as to the nature of "hysteria," is to be found in the frank adoption of the term "hysterical" in its most literal sense, and that in the future the uterus will be held to be in the main responsible for those various manifestations and disorders denominated "hysterical."—*Transactions of the International Medical Congress*, 1881.

## Figures and Tables

**Fig. I. f1:**
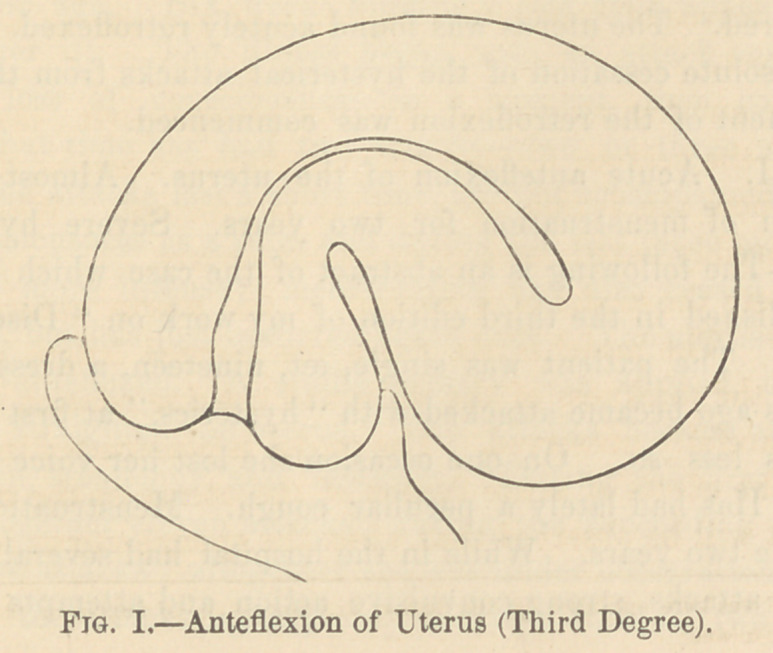


**Fig. II. f2:**